# The Higher Water Absorption Capacity of Small Root System Improved the Yield and Water Use Efficiency of Maize

**DOI:** 10.3390/plants11172300

**Published:** 2022-09-02

**Authors:** Minfei Yan, Li Zhang, Yuanyuan Ren, Tingting Zhang, Shaowei Zhang, Hongbing Li, Yinglong Chen, Suiqi Zhang

**Affiliations:** 1 State Key Laboratory of Soil Erosion and Dryland Farming on the Loess Plateau, Northwest A&F University, Yangling 712100, China; 2 College of Forestry, Northwest A&F University, Yangling 712100, China; 3 College of Traditional Chinese Medicine, Weifang Medical University, Weifang 261053, China; 4 Geography and Environmental Engineering Department, Baoji University of Arts and Sciences, Baoji 721013, China; 5 The UWA Institute of Agriculture, and UWA School of Agriculture and Environment, The University of Western Australia, Perth, WA 6001, Australia

**Keywords:** leaf water potential, root hydraulic conductivity, root size, water use efficiency, yield

## Abstract

The root system in plants absorbs water and minerals. However, the relationship among root size, yield, and water use efficiency (WUE) is controversial. Two pot experiments were conducted to explore these relationships by using two maize varieties with contrasting root sizes and reducing the root–shoot ratio (R/S) through root pruning to eliminate genotypic effects. Maize plants were grown in an open rainout shelter under both water-sufficient and deficient conditions. Yield-related parameters, root hydraulic conductivity (Lpr), and WUE were determined. The results showed that the small root variety (XY) has a higher yield and WUE compared to large root variety (QL) under both soil moisture conditions, likely related to the higher Lpr of XY. XY also had a higher leaf water potential than QL under drought stress, indicating that small root system could provide enough water to the shoot. Further pot experiment showed that both small and large root pruning on QL (cut off about 1/5 roots, RP1; and cut off about 1/3 roots, RP2, respectively) improved WUE and Lpr, and the RP1 yield increased by 12.9% compared to the control under well-watered conditions. Root pruning decreased transpiration and increased photosynthesis. Thus, this study reveals that it is possible to increase water absorption, yield, and WUE by reducing R/S in modern maize varieties, which may be important for the future breeding of new cultivars suitable for arid regions.

## 1. Introduction

Agriculture consumes approximately 70% of the global freshwater; thus, the increasing frequency and intensity of drought events associated with climate change are threatening water availability and crop yield [1]. Additionally, water availability is in jeopardy because of population expansion. A reduction in the overall agricultural water supply may be inevitable [2]; therefore, efficient utilization of limited water resources is essential for improving agricultural productivity.

Water use efficiency (WUE) reflects the relationship between plant productivity and water consumption and is regulated by the environment and the crop itself. Roots are the primary uptake sites for water and minerals. Recently, several studies have considered roots as central for improving yield and WUE of crops [3,4]. Thus, the root system is vital for sustaining crop productivity while restricting water consumption. Furthermore, the form and function of the root system are known to influence shoot physiology [5].

The size of the root system is determined by the total root biomass, cumulative length, and length density [6]. However, not all roots are capable of absorbing water and nutrients. An excessively large root system has limited benefits as it is assumed that the formation and maintenance of the system requires considerable amounts of photoassimilate [7]. The estimated amount of photoassimilate invested in one unit of root dry matter can produce two units of shoot dry matter [8]. Moreover, a large root system demands substantial amounts of carbon input, which results in carbon waste [9], rapidly depletes soil water, induces premature terminal drought, and lowers yield and WUE [10]. Root system efficiency is dependent on its ability to absorb water and nutrients and transport them to the shoot. It is also contingent on absolute and relative amounts of energy consumed by the shoot [5]. A large root system may be conducive to plant survival but not necessarily to agricultural production. Thus, excessively large root systems may not improve crop yield or WUE.

The relationships among the root size, crop yield, and WUE are controversial. Some studies recommend that drought-resistant varieties should have large root systems, suggesting root size as an indicator of drought resistance in crops [6,11]. In contrast, other studies found that root systems with small biomass and root length have been unintentionally selected in modern cultivars [12,13]. Passioura [14] and Zhu et al. [15] proposed that small root systems are beneficial in improving WUE and yield. Additionally, previous studies have shown that the WUE of wheat gradually improves with increasing ploidy, while the root–shoot ratio (R/S) decreases [16,17]. Recent studies have found that reduction of root biomass can enhance WUE of the crop [18], and yield of the large root variety was lower than that of the small root variety [19]. New understanding of the role that root system form and function play in crop adaptation is emerging. However, the relationship between root size and water absorption has rarely been investigated.

Water absorption capacity of the root system has an important influence on the water state of shoots and the normal growth and development of plants, which directly affects yield and WUE [20,21]. Root hydraulic conductivity (Lpr) reflects root ability to absorb water and represents water flux through the unit root surface area (or length) per unit of time and pressure gradient [22,23]. Lpr changes during different growth stages and environments [24]. In the crop evolution process, Lpr showed an increasing trend [25]. Lack of nutrients and water usually leads to a significant decrease in Lpr, but with restoration of the nutrient and water supply, it may rapidly increase [26,27]. It has been shown that Lpr significantly increased as the root biomass decreased in seedling stage under hydroponic conditions [28,29]. However, there are few reports on the relationship between root size and water absorption in soil; consequently, this relationship still needs to be clarified.

Herein, we studied the relationship between root size, crop yield, WUE, and water absorption using two varieties of maize with contrasting root sizes in a pot experiment. We hypothesized that small root maize (in terms of root biomass) would have a higher yield and WUE than the maize with an excessively large root system, related to the higher capacity of water absorption. This hypothesis was further tested by reducing the R/S through root pruning and eliminating the genotypic effects in the anther pot experiment. This study is of great significance for breeding new plant varieties with a high yield and efficient use of resources.

## 2. Results

### 2.1. Root Traits

**Figure 1:**
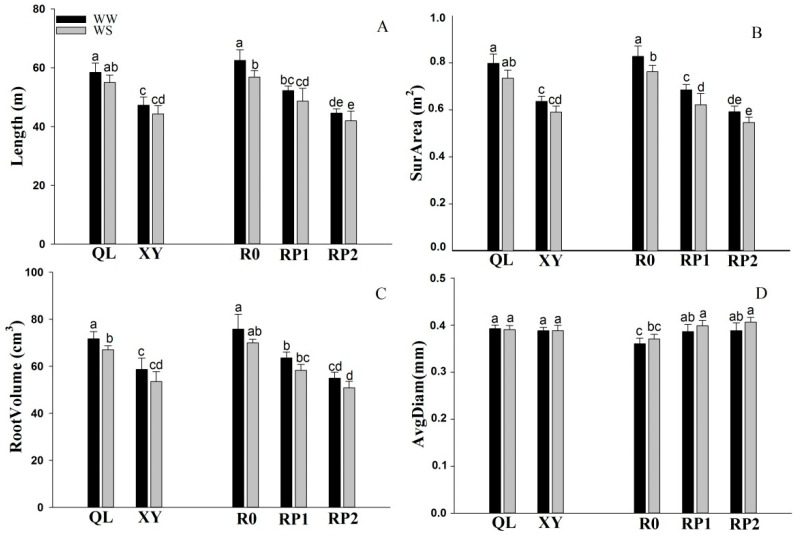
Total root length (**A**), root surface area (**B**), root volume (**C**), and average root diameter (**D**) under well-watered conditions (WW) and drought stress (WS) at jointing stage. QL (large root variety), XY (small root variety), R0 (no root pruning), RP1 (small root pruning) and RP2 (large root pruning). Values are means ± standard error (*n* = 3). Different letters indicate statistically significant differences (*p* < 0.05) after ANOVA and Duncan’s test.

The root biomass of the small-root variety (XY) was 18.43% and 23.57% lower than that of the large-root variety (QL) under well-watered conditions, at jointing and anthesis stages, respectively (Table 1). Furthermore, the root biomass of XY was 17.71% and 19.49% lower than that of QL under drought stress, at the jointing stage and anthesis stage, respectively (Table 1). XY exhibited a smaller R/S and shorter root length than QL. Additionally, root surface area and root volume were significantly lower in XY than those in QL (Figure 1A–C). There was no significant difference in root diameter between QL and XY under the two soil moisture conditions (Figure 1D). The shoot biomass of QL was significantly greater than that of XY at jointing stage, but they had no significant difference at anthesis stage.

Root pruning significantly reduced root biomass and R/S at the jointing stage. Under well-watered conditions, the root biomass of RP1 and RP2 was 18.46% and 28.51% lower than that of R0, respectively (Table 1). Furthermore, under drought stress, the root biomass of RP1 and RP2 was 18.15% and 29.65% lower than that of R0, respectively (Table 1). Root length, surface area, and volume exhibited the same trends as root biomass (Figure 1A–C). Average root diameter increased after root pruning and drought treatment (Figure 1D). Nevertheless, root pruning had no effect on shoot biomass.

### 2.2. Root Hydraulic Conductivity

**Figure 2:**
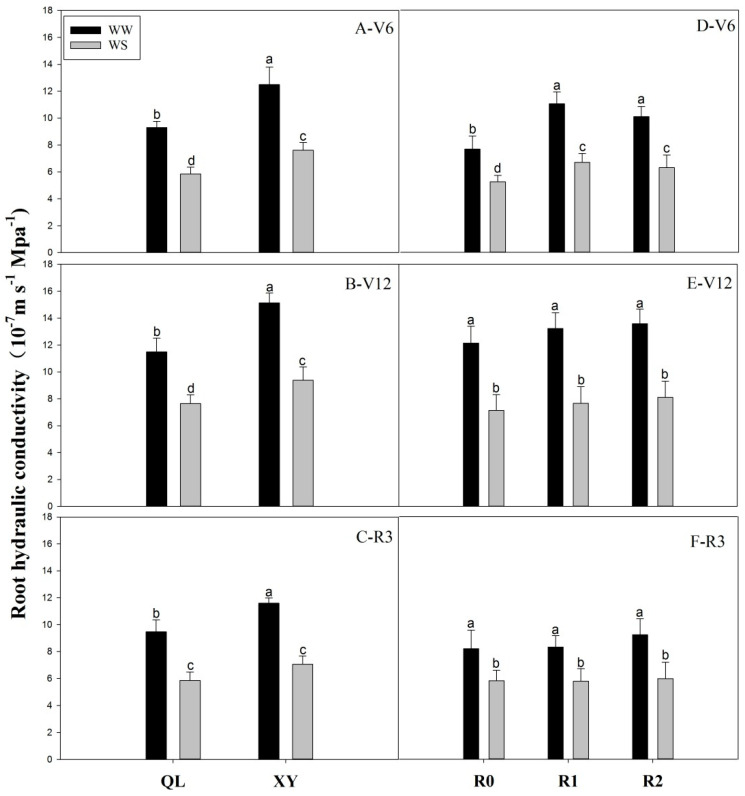
Lpr in Experiment 1 at jointing stage (**A**−V6), anthesis stage (**B**−V12), and milk stage (**C**−R3); Lpr in Experiment 2 at jointing stage (**D**−V6), anthesis stage (**E**-V12), and the milk stage (**F**−R3). Values are means ± standard error (*n* = 6). Different letter indicates significant differences among treatments (*p* < 0.05) based on Duncan’s test. The treatment abbreviations are defined in Figure 1.

Lpr for XY was 34.32%, 31.66%, and 22.37% higher than that of QL under well-watered conditions at jointing, anthesis and the milk stages, respectively. And was 30.13%, 22.75%, and 20.61% higher under drought stress, respectively (Figure 2A–C). Root pruning significantly enhanced the Lpr of maize at the jointing stage. At the jointing stage, the Lpr of RP1 and RP2 was 43.90% and 31.54% higher than that of the control under well-watered conditions, respectively, and 27.37% and 19.79% higher respectively, under drought stress (Figure 2D). Drought stress significantly inhibited Lpr, with a value 31.54% lower compared to that of the control (Figure 2D). The Lpr of pruned plants was not significantly different from that of the control at anthesis and milk stages (Figure 2E,F).

### 2.3. Leaf Water Potential (Ψ_leaf_)

**Figure 3:**
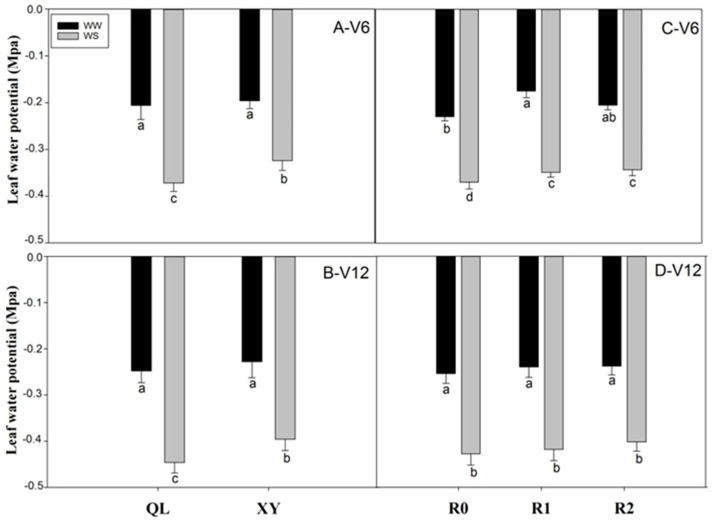
Leaf water potential at the jointing stage (**A**−V6) and at anthesis stage (**B**−V12) in Experiment 1. Leaf water potential at the jointing stage (**C**−V6) and at anthesis stage (**D**−V12) in Experiment 2. Values are means ± standard error (*n* = 5). Different letters indicate significant differences among treatments (*p* < 0.05) based on Duncan’s test. The treatment abbreviations are defined in Figure 1.

Under well-watered conditions, there was no significant difference between QL and XY in terms of Ψ_leaf_ at the jointing and anthesis stages. However, under drought stress, the Ψ_leaf_ of XY was 14.81% and 12.63% higher than that of QL at the jointing and anthesis stages, respectively (Figure 3A,B). Root pruning increased Ψ_leaf_ in maize at jointing stage. The Ψ_leaf_ of RP1 and RP2 was 23.9% and 10.87% higher than that of R0 under well-watered conditions, respectively, and 5.71% and 7.25% higher than that of R0 under drought stress at jointing stage, respectively (Figure 3C). However, there was no obvious difference in Ψ_leaf_ at the anthesis stage (Figure 3D).

### 2.4. Leaf Gas Exchange Parameters

**Figure 4:**
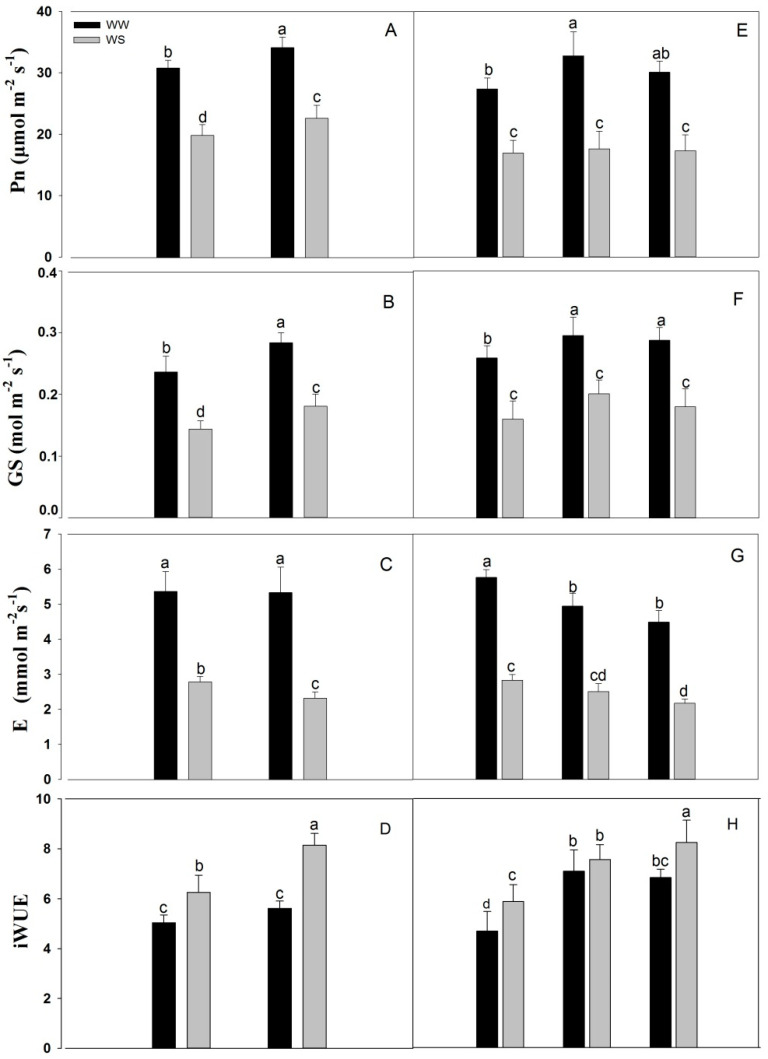
Photosynthetic rate (Pn) (**A**), stomatal conductivity (GS) (**B**), transpiration rate (E) (**C**) and instantaneous leaf water use efficiency (iWUE) (**D**) in Experiment 1 at the jointing stage. Pn (**E**), GS (**F**), E (**G**) and iWUE (**H**) in Experiment 2 at the jointing stage. Values are means ± standard error (*n* = 6). Different letters indicate significant differences among treatments (*p* < 0.05) based on Duncan’s test. The treatment abbreviations are defined in Figure 1.

Under well-watered conditions, the photosynthetic rate (Pn) and stomatal conductance (GS) of XY were 10.83% and 19.97% higher than those of QL, respectively (Figure 4A,B). There were no significant differences between XY and QL in terms of the transpiration rate (E) and instantaneous leaf water use efficiency (iWUE) under well-watered conditions (Figure 4C,D). Under drought stress, Pn, GS, and iWUE of XY were 13.90%, 25.80%, and 30.03% higher than those of QL, respectively (Figure 4A,B,D). The transpiration rate of XY was 16.73% lower than that of QL under drought stress (Figure 4C).

Root pruning increased the Pn in maize. Under well-watered conditions, the Pn of RP1 and RP2 was 19.66% and 9.90% higher than that of R0, respectively (Figure 4E), and the GS of RP1 and RP2 was 14.02% and 11.04% higher than that of R0, respectively (Figure 4F). However, under drought stress, there were no significant differences between the pruned plants and the control in terms of Pn and GS (Figure 4E,F). Overall, root pruning significantly reduced the relative E. Under well-watered conditions, the E of RP1 and RP2 was 14.23% and 22.18% lower than that of R0, respectively, while under drought stress, it was 11.52% and 23.22% lower, respectively (Figure 4G). Under well-watered conditions, the iWUE of RP1 and RP2 was 51.38% and 45.90% higher than that of R0, respectively, and was 28.38% and 40.06% higher than that of R0 under drought stress, respectively (Figure 4H).

### 2.5. Grain Yield (GY) and WUE

Table 2 shows that under well-watered conditions, the grain yield (GY), 100-kernel weight (HKW), ear length (EL), and harvest index (HI) of XY were 17.55%, 9.08%, 11.72%, and 5.56% higher than those of QL, respectively. Under drought stress, the GY, HKW, EL, and HI of XY were 18.27%, 11.42%, 9.68%, and 6.0% higher than those of QL, respectively.

Under well-watered conditions, the GY, HKW, and HI of RP1 increased by 12.87%, 6.35%, and 7.55%, respectively. Under drought stress, root pruning had no significant effect on GY, EL, or HI, but increased HKW of RP1 and RP2 by 9.30% and 12.22%, respectively.

Water significantly increased GY, HKW, EL, and HI (*p* < 0.05). Root size was significant associated with GY, HKW, and HI (*p* ≤ 0.05). However, there was no significant interaction between W and R in terms of HKW, EL, and HI (*p* > 0.05) (Table 2).

**Figure 5:**
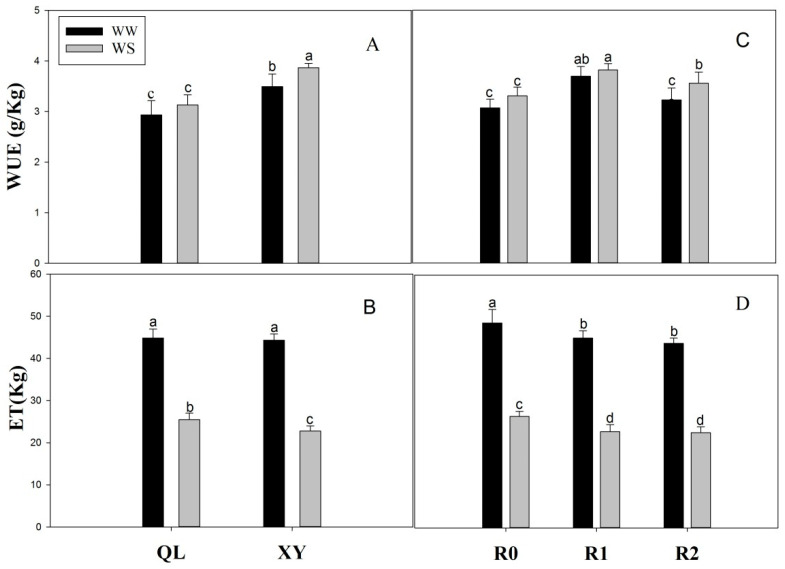
Water use efficiency (WUE) (**A**), evapo-transpiration (ET) (**B**) (irrigation throughout the growth period) in Experiment 1. WUE (**C**) and ET (**D**) in Experiment 2. Values are means ± standard error (*n* = 6). Different letters indicate significant differences among treatments (*p* < 0.05) based on Duncan’s test. The treatment abbreviations are defined in Figure 1.

The WUE of XY was 19.14% and 24.45% higher than that of QL under well-watered and drought stress conditions, respectively (Figure 5A). Under well-watered conditions, there was no significant difference between XY and QL in terms of ET. However, the ET of XY was 10.59% lower than that of QL under drought stress (Figure 5B).

Root pruning significantly increased WUE. Under well-watered conditions, RP1 and RP2 increased by 20.32% and 5.00%, respectively. Under drought stress, RP1 and RP2 increased by 15.46% and 7.63%, respectively (Figure 5C). Additionally, root pruning reduced maize water consumption throughout the entire growth period. Under well-watered conditions, the ET decreased in RP1 and RP2 by 7.40% and 14.64%, respectively. Under drought stress, the ET decreased in RP1 and RP2 by 9.96% and 11.61%, respectively (Figure 5D).

### 2.6. Principal Component Analysis (PCA) of Maize Growth and Physiological Parameters

Correlation analyses revealed that the HKW, EL, ET, Lpr, Ψ_leaf_, leaf gas parameters, and above-ground dry weight at maturity (SW) significantly affected GY. Their correlation coefficients were 0.796, 0.692, 0.903, 0.701, 0.892, 0.885, 0.857, 0.786, and 0.912, respectively (Table 3). Lpr was significantly positively correlated with Ψ_leaf_, E, HKW, and SW. Pn was significantly positively correlated with Ψ_leaf_, Lpr, SW, and E.

**Figure 6:**
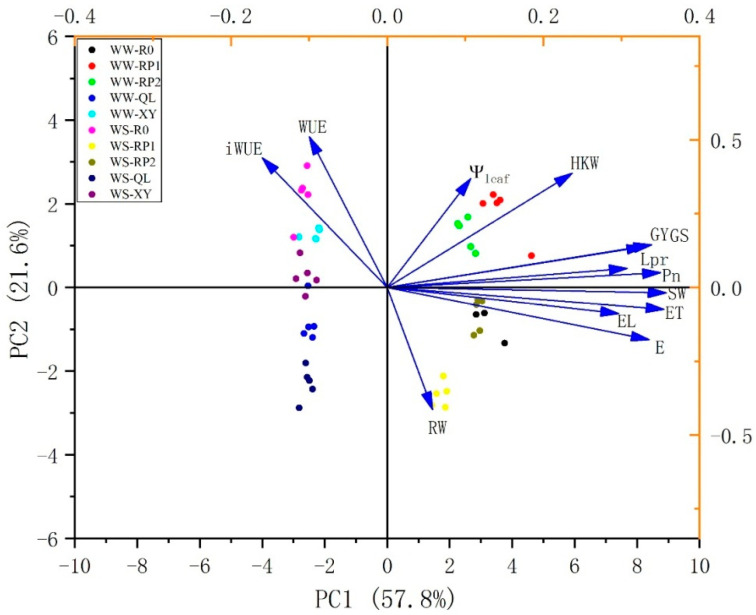
PCA of growth and physiological traits under water deficit treatment. WW, well-watered; WS, drought stress.

A PCA was conducted to identify the key parameters responsible for the response patterns of the roots of various sizes to maize growth. Morphophysiological data were used to disclose the differences and similarities among treatments. The first and second principal components (PC1 and PC2) accounted for 57.8% and 21.6% of the total variation, respectively (Figure 6). PC1 separated the effects of water deficit. GY, ET, EL, Lpr, Pn, GS, SW, and E contributed to PC1 while HKW, Ψ_leaf_, iWUE, RW (root weight at jointing stage), and WUE contributed to PC2 (Table S1).

## 3. Discussion

There is presumably an optimal R/S at which grain yield is maximal [14]. Here, root biomass of XY (small roots) was significantly smaller than that of QL (large roots) during the entire maize growth period. The roots total length, surface area, and volume were significantly smaller in XY than in QL (Figure 1 and Table 1). Root pruning significantly reduced the maize R/S. Hence, we successfully simulated a maize cultivar with a relatively small R/S. Both root pruning and drought stress increased root diameter (Figure 1D); this may be because that with the increase of root diameter, the area of water absorption of the root system also increases and thus it contributes to the absorption of soil water.

The Lpr reflects the ability of roots to absorb water [22], and its regulation plays an important role in maintaining water status of the entire plant. Lpr also changes in different growth stages and growing environments [28]. Additionally, it varies in different species or varieties of the same species. Moreover, Lpr was negatively correlated with the root surface area and length [25]. In maize and wheat, Lpr increased with a decreasing R/S [28,29,30]. We also observed that Lpr was significantly higher in XY than in QL under both soil moisture conditions and throughout the maize growth period (Figure 2A–C). Thus, small roots do not have lower water absorption capacities than large roots. Our subsequent root-pruning experiment validated this hypothesis. Root pruning reduced root biomass and significantly increased Lpr, as compared with unpruned plants at the jointing stage (Figure 2D). Vysotskaya et al. [28] also revealed an increase in hydraulic conductivity of the root system following partial root excision. Moreover, there was no significant change in root biomass after compensatory growth at anthesis and milk stages in our study, and Lpr of pruned plants was not significantly different (*p* > 0.05) from that of the control at the anthesis and milk stages (Figure 2E,F). Root pruning leads to a reduced R/S during the jointing stage; however, there is a control mechanism balancing growth of the above- and below-ground plant parts [31]. This mechanism enables plants to restore their R/S ratio after root pruning. In this study, there was no significant difference in the R/S and Lpr between pruned plants and the control at anthesis and milk stages, respectively. Additionally, Lpr decreased after anthesis (Figure 2), which may be due to the decreased root activity after anthesis [32]. Under water deficiency conditions, the Lpr extremely limited the water uptake and transport. Lpr decreased under drought stress [33,34], and the same result was observed in our experiment (Figure 2).

Changes in Lpr, in turn, are known to affect water potential in plants [35], and there was a significant positive correlation between Lpr and leaf water potential [36]. Leaf water potential reflects the water state of plant and the adaptability of plants to the environment [37]. It can be reasonably assumed that small roots should have less water supply capacity to aboveground than large roots, resulting in a lower leaf water potential, while in this study, the leaf water potentials of the plants with small roots were not lower than those for the plants with large roots (Figure 3). This observation may be explained by the ability of the small root systems to transport sufficient water to the shoots. A previous study has also reported that the only remaining root was capable to supply the shoot with water [28]. The resistance of the root system to water transport is greater than that of the shoot, so the Lpr is the main factor that determines the water potential of the aboveground [38]. Therefore, the increase of Lpr in small root plants was the main factor for the high water potential of shoots. Furthermore, plants with small roots might have relatively lower foliar transpiration rates than those with large roots (Figure 4). Moreover, under drought conditions, the leaf water potential of small-root system XY was higher than that of large-root system QL at jointing and anthesis stages, indicating that XY with better drought resistance can maintain a better water balance under drought stress.

Roots play a vital role in water uptake, and thus affects the photosynthesis, which is associated with changes in yield [39,40]. A previous study showed that crop photosynthetic rates increased with a decreasing R/S [41,42]. In the present study, the photosynthetic rate of XY was 10.83% higher than that of QL (Figure 4A). Root pruning also increased the photosynthetic rate in maize (Figure 4E), and enhanced photosynthesis may also contribute to a high yield in small-root maize. Fang et al. [41] reported that root pruning lowered the R/S during early growth stage, but increased rate of flag leaf photosynthesis at anthesis. Photosynthesis compared with the controls during early growth, with higher photosynthesis in the later growth circle, showed that root pruning enhanced plant growth [42]. Drought stress severely affects photosynthesis and stomatal characteristics [43,44], as chlorophyll biosynthesis was blocked and photosynthetic rates were lowered in certain crops [44,45]. In our study, the photosynthetic rate had decreased under drought stress (Figure 4A,E). Furthermore, stomatal conductance and the photosynthetic rate showed a consistent change trend (Figure 4B,F), and the same results have been reported in a previous study [8]. Moreover, plant hydraulic conductance has been reported to be more relevant in regulating stomatal conductance, resulting in minimal variations in leaf water potential [46,47]. There were no differences in the transpiration rate between XY and QL under well-watered conditions; however, the transpiration rate of XY was lower than QL under drought conditions (Figure 4C). This may also be a strategy for XY to improve drought resistance and maintain the water balance of plants. Nevertheless, root pruning lowered the transpiration rate under both moisture conditions, likely due to root pruning breaking the original water balance in plants. Reducing the transpiration rate through a series of physiological adjustments may be a strategy for plants to re-maintain water balance. The stomata are highly sensitive to water deficit. They rapidly respond to changes in water content and availability and enable plants to balance water loss and uptake [48]. Therefore, plants with pruned roots have a lower transpiration rate than those with intact roots (Figure 4G). Hence, small roots may reduce water consumption by lowering transpiration rate and maintaining water balance in the plant.

Recent studies have shown that selection varieties seem to unknowingly increase WUE and grain yield by gradually selecting smaller root systems [13,49,50]. Further breeding is required to improve root traits and yield potential. Previous studies have also found that plants with small roots have higher yield than those with large roots [42,43,51]. We discovered that XY and pruned plants had a significantly higher grain yield than QL and unpruned plants; also, 100-kernel weight and ear length are important grain yield components [52], and their elevation accounted for the relatively high grain yield of XY and pruned plants (Table 2). Roots are the main reservoirs for substances absorbed from the substrate and require twice as much photosynthate as buds for dry matter production [9]. Moreover, root system maintenance demands more energy than its construction [21]. Over 50% of all photosynthate is lost through root respiration [53]. A reduction in the metabolic cost of root growth could enhance water access and productivity in the plant by increasing availability of metabolic resources required for nutrient uptake, growth, and reproduction [54]. Although aboveground interactions are important, much of the competition for soil resources, such as nutrients and water, takes place belowground [42]. Thus, large roots are not absolutely essential, and their reduction may increase the amount of photosynthate available for new shoots and GY. Hence, plants with small roots have higher GY than those with large roots. This proves that the large root system is not the symbol for high yield, as the small root system can also have a relatively high yield; therefore, the size of the root system cannot be used as an indicator for identifying high yield. Furthermore, in this study, small-root plants had a higher yield, but used less water (ET) (Figure 5B,D). Consequently, this confirms that plants with small roots have a relatively higher WUE, especially under drought stress (Figure 5A,C), which correlates with previous research [18]. Some studies have suggested that the root system is an important index to determine crop WUE [55,56]. Our study showed that the size of the crop root system strongly influences WUE. The results of this study support the idea that breeding for drought resistance, enhanced GY, and improved WUE in arid and semiarid areas should not focus exclusively on developing cultivars with large roots.

PCA of the datasets revealed differences and similarities between plants with different root sizes subjected to various water conditions. PC1 separated maize with different root sizes planted under normal and drought conditions. The key factors in PC1 were GY, ET, EL, Lpr, Pn, GS, SW, and E (Table S1). Therefore, water absorption, photosynthesis, and dry matter accumulation varies among crops with different root sizes. The grain yield from XY and RP1 were higher than QL and R0 under the two soil moisture conditions. In these indices, improved 100-kernel weight and ear length were considered two of the most likely approaches to improve yield potential.

## 4. Material and Methods

### 4.1. Plant Materials and Experimental Design

#### 4.1.1. Experiment 1

A pot experiment was conducted between May and September 2018 at Yangling, China (108.07° E, 34.27° N). Seeds of maize (*Zea mays* L.) cultivars with large root biomass (var. Qinlong 14) and small root biomass (var. Xianyu 335) [57,58] were disinfected with 2% (*w*/*v*) sodium hypochlorite. Seeds were sprouted in a dark germination chamber at 28 °C. After 3 d, the sprouts were sown in plastic pots (H = 27 cm; D = 28 cm). Each pot was filled with 17 kg sieved loamy clay (22% field water capacity (FWC)), collected from the top 0–20 cm of cropland at Yangling. The soil texture parameters are shown in Table 4. Each pot was fitted with a polyvinyl chloride tube with a diameter of 1.5 cm (for irrigation). All pots were supplied with 200 mg/kg CH_4_N_2_O and 150 mg/kg KH_2_PO_4_. Two seeds were sown in each pot and then thinned to one seedling per pot after 7 days. Each treatment was replicated in 16 pots. At the five-leaf stage, plants were either well-watered (WW; 75–85% FWC) or subjected to drought stress (WS; 35–45% FWC). Drought stress was exerted through stopping the water supply to reach 35 ± 5% FWC, followed by irrigation. The relative soil water content was regulated based on soil weight. The pots were weighed and re-watered at 6:00 p.m. daily.

#### 4.1.2. Experiment 2

**Figure 7:**
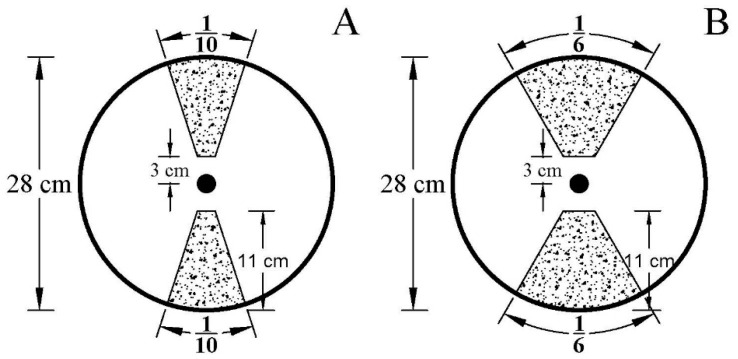
Root pruning method of small root pruning (**A**) and large root pruning (**B**). The root system was cut off vertically from the soil surface to the bottom along the two sides, approximately 3 cm away from the plant using a 28 cm single-sided knife at the jointing stage.

A pot experiment was conducted between May and September 2019 at Yangling, China. The Zea mays L. var. Qinlong 14 with large root biomass was used. The experimental planting method was the same as that used for Experiment 1. The well-watered (WW; 75–85% FWC) and drought stress (WS; 35–45% FWC) treatments were initiated at the five-leaf stage. At the maize six-leaf stage, the plants were then subjected to the following root pruning treatments: (1) small root pruning (RP1) (cut off about 1/5 root system); (2) large root pruning (RP2) (cut off about 1/3 root system); (3) no root pruning (R0) (control). Root system was cut off from the soil vertically from the soil surface to the bottom along the two sides, and approximately 3 cm away from the plant using a 28 cm single-sided knife, with the assigned percentage area for RP1 and RP2, as illustrated in Figure 7.

### 4.2. Measurements

#### 4.2.1. Root Hydraulic Conductivity and Leaf Water Potential Measurements

A high-pressure flow meter (HPFM-Gen3; Dynamax Inc., Houston, TX, USA) was used to determine the Lpr. Seedlings were excised at the first internodes, a pressure coupler was connected to the incision site, and the air was evacuated. The measurement range was determined, and the instantaneous method was used for the measurements. The pressure was increased to ~300 MPa at a rate of 2–5 MPa s^−1^ and the relationship between flow velocity pressure and time was determined. Roots were selected, rinsed, and scanned, and their surface areas were measured. Samples were taken at the jointing, anthesis and milk stages respectively, and six replicates were measured. Lpr was calculated as follows:

Lpr = V × S^−1^ × p^−1^ × t^−1^ (1)

where Lpr is the root hydraulic conductivity (m s^−1^ MPa^−1^), V is the total volume of water passing through the root (m^3^), S is the root surface area (m^2^), p is the external pressure (MPa), and t is time (s).

Leaf water potential was measured in a pressure chamber (Type 3005; Soil Moisture Equipment, Santa Barbara, CA, USA) before dawn at the jointing and anthesis stages.

#### 4.2.2. Leaf Gas Exchange Parameters

The Pn, GS, and E were measured between 9:30 a.m. and 11:00 a.m. at the centers of the newest, fully expanded leaves with a portable photosynthesis system (Li-6800; LI-COR, Inc., Lincoln, NB, USA) under 1200 μmol m^−2^ s^−1^ light intensity, 60% relative humidity, and 400 μmol mol^−1^ CO_2_. Data were collected at jointing stage, and there were six replicates. Instantaneous iWUE was calculated as follows:

iWUE = Pn/E(2)

where iWUE is the instantaneous leaf water use efficiency, Pn is the photosynthetic rate (µmol m^−2^ s^−1^), E is the transpiration rate (mmol m^−2^ s^−1^).

#### 4.2.3. Root Sampling and Measurements

Excised and residual roots were selected (cut-off root system distinguished from normal growing root system by color at the anthesis and milk stages; cut-off root system is gray-black; normal growing root system is white), rinsed, and scanned using a scanner (Epson Perfection V800, Seiko Epson Crop., Suwa, Japan), with a transparency adapter at 300 dpi. Total root length (RL, cm), root surface area (RSA, cm^2^), and average root diameter (ARD, mm) were measured or calculated with an analysis software (WinRHIZO, Regent Instrument Inc., Québec, QC, Canada). Root samples were collected in triplicate at the jointing, anthesis, and milk stages, oven-dried at 75 °C for 48 h and weighed on an analytical balance.

#### 4.2.4. Water Use Efficiency (WUE) and Harvest Index (HI)

Whole plants were harvested at maturity, and the grain yield (GY), 100-kernel weight (HKW), ear length (EL), harvest index (HI), and WUE were determined by

HI = GY/SW(3)

where GY is the grain yield per pot at maturity; and SW is the aboveground biomass yield per pot at maturity, and

WUE (g/Kg) = GY/ET(4)

where GY is the grain yield per pot (g) and ET is the recorded total water consumption per pot over the entire growth cycle, which is measured by weight (Kg).

### 4.3. Statistical Analysis

The data were checked for normal distribution and equal variance, then analyzed with one-way ANOVA and Duncan’s multiple range tests, and their interactions at *p* ≤ 0.05 using SPSS v.14.0 (SPSS Inc., Chicago, IL, USA). Correlations were examined using Pearson’s correlation coefficient. SigmaPlot v. 12.5 (Systat Inc., San Jose, CA, USA) was used to correlate and plot indicators. A principal component analysis (PCA) was performed on all measured physiological parameters in Origin v. 2018 (OriginLab, Northampton, MA, USA).

## 5. Conclusions

The results of this study demonstrate that leaf water potential of plants with small roots is not lower than that with large roots, which proves that small roots can transfer enough water to the ground.

The varieties with small root systems lead to a higher grain yield and WUE than those with excessively large root systems, which may be related to the higher capacity of water absorption. This suggests that drought-resistant breeding should not be limited to offspring with large roots and that appropriate R/S is the future direction of drought-resistant breeding.

## Figures and Tables

**Table 1:** Root dry weight, shoot dry weight and root/shoot ratio (R/S), with average values at jointing stage (V6) and anthesis stage (V12).

Treatment	Items	Pot Experiment 1				Pot Experiment 2					
		V6		V12		V6			V12		
		QL	XY	QL	XY	R0	RP1	RP2	R0	RP1	RP2
WW	Root (g)	24.53 ± 1.59 a	20.01 ± 1.44 b	39.42 ± 2.47 a	30.13 ± 3.48 b	26.38 ± 1.51 a	21.51 ± 1.14 b	18.86 ± 1.28 c	41.06 ± 1.91 a	38.62 ± 1.77 ab	36.53 ± 0.71 b
	Shoot (g)	64.14 ± 4.11 a	54.15 ± 1.84 bc	129.61 ± 7.91 a	119.20 ± 9.01 a	58.58 ± 1.57 a	56.40 ± 3.05 a	56.05 ± 2.05 a	127.74 ± 15.38 a	131.42 ± 10.37 a	125.02 ± 3.48 a
	R/S	0.38 ± 0.03 b	0.34 ± 0.01 c	0.31 ± 0.03 c	0.26 ± 0.05 d	0.45 ± 0.01 a	0.38 ± 0.02 b	0.33 ± 0.03 c	0.32 ± 0.03 bc	0.29 ± 0.02 c	0.29 ± 0.01 c
WS	Root (g)	22.98 ± 1.23 a	18.91 ± 1.56 b	29.29 ± 1.40 ab	23.58 ± 1.65 b	24.89 ± 0.75 a	20.37 ± 0.40 b	17.51 ± 0.71 c	28.62 ± 2.03 c	26.22 ± 0.67 cd	24.15 ± 0.93 d
	Shoot (g)	59.36 ± 2.87 ab	52.16 ± 1.87 c	70.84 ± 7.48 b	67.12 ± 2.32 b	53.81 ± 1.17 b	52.19 ± 3.17 b	51.73 ± 1.80 b	78.75 ± 2.70 b	76.03 ± 13.36 b	74.53 ± 8.17 b
	R/S	0.43 ± 0.02 a	0.36 ± 0.01 b	0.42 ± 0.06 a	0.35 ± 0.03 b	0.47 ± 0.02 a	0.39 ± 0.02 b	0.34 ± 0.02 c	0.39 ± 0.02 a	0.35 ± 0.05 b	0.30 ± 0.01 bc

QL (large root variety), XY (small root variety), R0 (without root pruning), RP1 (small root pruning) and RP2 (large root pruning) under well-watered (WW) and drought stress (WS). Values are means ± standard error (*n* = 3). Different letters indicate significant differences among treatments (*p* < 0.05) based on Duncan’s test.

**Table 2:** Yield and yield-related components under well-watered (WW) and drought stress (WS) conditions.

Treatment	Grain Yield (g/pot)	100-Kernel Weight (g)	Ear Length (cm)	HI
WW				
QL	131.56b ± 7.31	29.18 ± 0.84 ab	15.62 ± 1.02 b	0.54 ± 0.03 b
XY	154.65 ± 5.88 a	31.83 ± 1.31 a	17.45 ± 0.38 a	0.57 ± 0.05 a
R0	148.01 ± 4.67 b	36.51 ± 0.92 b	15.58 ± 1.79 a	0.53 ± 0.01 b
RP1	167.06 ± 2.47 a	38.83 ± 0.89 a	16.62 ± 0.99 a	0.57 ± 0.05 a
RP2	143.88 ± 8.73 b	36.75 ± 0.84 b	15.07 ± 1.74 a	0.55 ± 0.02 b
WS				
QL	74.43 ± 2.40 d	25.83 ± 0.90 d	12.81 ± 0.88 d	0.50 ± 0.02 c
XY	88.03 ± 3.90 c	28.78 ± 1.86 c	14.05 ± 1.07 c	0.53 ± 0.02 b
R0	85.90 ± 1.15 c	29.12 ± 0.64 d	9.50 ± 1.12 b	0.47 ± 0.01 c
RP1	89.52 ± 2.78 c	31.83 ± 0.41 c	10.66 ± 1.83 b	0.48 ± 0.02 c
RP2	83.77 ± 2.38 c	32.68 ± 2.19 c	10.87 ± 0.95 b	0.46 ± 0.03 c
Probability level of ANOVA				
W	**	**	*	**
R	**	*	NS	**
W × R	*	NS	NS	NS

QL (large root variety), XY (small root variety), R0 (no root pruning), RP1 (small root pruning), and RP2 (large root pruning) under well-watered (WW) and drought stress (WS) conditions. Values are the means ± standard error (*n* = 6). Different letters indicate significant differences among treatments (*p* < 0.05) based on Duncan’s test. ANOVA results for the main factors (water, W; root size, R) and their interactions (W × R) are given for each parameter. Symbology: *, *p* < 0.05; **, *p* < 0.01; NS, not significant.

**Table 3:** Correlations among yield, yield components, and physiological indices under well-watered (WW) and drought stress (WS) conditions.

	HKW	EL	WUE	ET	Lpr	Ψ_leaf_	Pn	GS	E	iWUE	RW	SW
GY	0.796 **	0.692 **	0.731 *	0.903 **	0.701 **	0.892 **	0.885 **	0.857 *	0.786 *	−0.301 *	0.126	0.912 **
HKW		0.319 *	0.321 *	0.623 **	0.431 **	0.638 **	0.625 **	0.724	0.463	0.153	−0.126	0.588 **
EL			−0.338	0.743 **	0.211	0.414	0.760 **	0.640 *	0.472	−0.327 *	0.332	0.530
WUE				−0.398 **	0.692 *	0.242	−0.206	−0.551	−0.474 **	0.655 **	−0.493 *	−0.285 *
ET					0.689 **	0.614 *	0.584	0.839 *	0.932 **	−0.575 **	0.348 *	0.838 **
Lpr						0.851 **	0.642	0.570	0.662 **	−0.150	−0.218	0.745 **
Ψ_leaf_							0.563	−0.861 *	−0.847 **	0.357 *	−0.063	0.198
Pn								0.864 **	0.835 **	−0.263	0.068	0.835 **
GS									0.728 *	−0.264	0.028	0.769 **
E										−0.702 **	0.419 **	0.778 **
iWUE											−0.741 **	−0.434

GY, grain yield; HKW, 100-grain weight; EL, ear length; WUE, water use efficiency; ET, evapo-transpiration; Lpr, root hydraulic conductivity; Ψ_leaf_, leaf water potential; Pn, photosynthetic rate; GS, stomatal conductivity; E, transpiration rate; iWUE, instantaneous leaf water use efficiency; RW, root dry weight at jointing stage; SW, above-ground dry weight at maturity. Symbology: **, significant correlation at the 0.01 level; *, significant correlation at the 0.05 level.

**Table 4:** The soil texture parameters.

Texture	PH	Bulk Density (g/cm^3^)	Available N (mg/kg)	Available p (mg/kg)	Available K (mg/kg)	Organic Matter (g/kg)	Total N (mg/kg)
Loam	7.6	1.37	14.22	13.8	124.6	0.92	18.0

## Data Availability

The data that support the findings of this study are available in the main text and the supplementary materials.

## Supplementary Materials

The following supporting information can be downloaded at: https://www.mdpi.com/article/10.3390/plants11172300/s1, Table S1: PCA of growth and physiological parameters under well-watered (WW) and drought stress (WS) conditions.
