# Supplementary material for: The Higher Water Absorption Capacity of Small Root System Improved the Yield and Water Use Efficiency of Maize
Source: Plants (Basel). 2022 Sep 2;11(17):2300. doi: 10.3390/plants11172300 (PMC9460845; doi:10.3390/plants11172300)
Supplement: Supplementary file 1 [file plants-11-02300-s001.zip › plants-1796232-supplementary.pdf]

Supplementary Material

Table S1. PCA of growth and physiological parameters under well-watered (WW) and drought stress (WS) conditions. Means for both water treatments were combined for the PCA.

	**PC1**	**PC2**
GY	**0.3397**	0.1422
HKW	0.2418	**0.3792**
EL	**0.2921**	-0.0862
WUE	-0.1033	**0.5153**
ET	**0.3551**	-0.0738
Lpr	**0.2948**	0.0851
Ψ	0.1173	**0.3584**
Pn	**0.3436**	0.0640
GS	**0.3285**	0.1497
E	**0.3355**	-0.1751
iWUE	-0.1751	**0.4366**
RW	0.0834	**-0.4168**
SW	**0.3563**	-0.0103
	Variation proportion	
Eigenvalue	7.51422	2.6941
Variance (%)	57.8017	20.7242
Cumulative variability (%)	57.8017	78.5259

Abbreviations for the indices are defined in Table 3. Numbers in bold font indicate the key PC factors of PC with the highest scores.
